# Phase I first-in-human study of HLX07, a novel and improved recombinant anti-EGFR humanized monoclonal antibody, in patients with advanced solid cancers

**DOI:** 10.1007/s10637-021-01099-1

**Published:** 2021-03-13

**Authors:** Ming-Mo Hou, Ching-Liang Ho, Hsuan-Yu Lin, Yunting Zhu, Xiaodi Zhang

**Affiliations:** 1 Department of Medical Oncology, Chang Gung Memorial Hospital/Chang Gung University, Taoyuan City, Taiwan; 2 Division of Hematology/Oncology, Tri-Service General Hospital, National Defense Medical Center, Taipei, Taiwan; 3 Department of Internal Medicine, Changhua Christian Hospital, Changhua City, Taiwan; 4 Shanghai Henlius Biotech, Inc., Shanghai, China

**Keywords:** Monoclonal antibody, Pharmacokinetics, Solid tumors, Toxicity, Efficacy

## Abstract

*Purpose* This study aimed to evaluate the safety and pharmacokinetic (PK) profiles of HLX07, a novel, recombinant, humanized anti-epidermal growth factor receptor (EGFR) antibody, in patients with advanced solid cancers who had failed standard therapy or for whom no standard therapy was available. *Methods* In this prospective, open-label, Phase I dose escalation study, patients aged ≥18 years (≥20 years for patients in Taiwan) with histologically-confirmed metastatic or recurrent epithelial carcinoma that had no *K-RAS* or *B-RAF* mutations were enrolled in a ‘3 + 3’ escalation design. HLX07 was administered weekly by 2-h intravenous infusion at doses ranging from 50 to 800 mg. The primary endpoint was summary listing of participants reporting treatment-emergent adverse events (TEAEs). Secondary endpoints included PK analysis, serum anti-HLX07 antibody assessments and efficacy. *Results* In total, 19 patients were enrolled between 1 October 2016 and 16 July 2019 to receive HLX07 at doses of 50 (n = 3), 100 (n = 3), 200 (n = 3), 400 (n = 3), 600 (n = 3) and 800 (n = 4) mg per week. All patients experienced at least one TEAE, most commonly fatigue (68.4%), nausea (47.4%), paronychia (31.6%) and vomiting (31.6%). Serious TEAEs were reported in 11 patients but only one serious TEAE (dyspnea in 600 mg cohort) was regarded as possibly related to study treatment. No dose limiting toxicity (DLT) was reported. Systemic exposure to HLX07 increased proportionally with dose. Anti-HLX07 antibodies were not detected in any patients. *Conclusion* HLX07 was well tolerated (at dose levels up to 800 mg/week) and promising in patients with advanced solid cancers.

**Clinical Trial Registration**: The study was registered at ClinicalTrials.gov: NCT02648490 (Jan 7, 2016).

**Supplementary Information:**

The online version contains supplementary material available at 10.1007/s10637-021-01099-1.

## Introduction

Epidermal growth factor receptor (EGFR), a transmembrane receptor belongs to the family of human epidermal growth factor receptor (HER), is involved in multiple signaling pathways including cell proliferation, motility, angiogenesis, apoptosis, autophagy and energy metabolism [1, 2]. EGFR has an expression rate of 47–100% in human solid tumors and is therefore an attractive target for anti-cancer drugs [3]. A number of EGFR-targeted drugs have been approved for clinical use [4]. The currently approved EGFR monoclonal antibodies (mAbs) are cetuximab, panitumumab, nimotuzumab and necitumumab [5–8].

Anti-EGFR mAbs are recommended by most international guidelines for the treatment of metastatic colorectal cancer [9, 10] as well as head and neck cancer [11, 12]. Of the approved anti-EGFR mAbs, cetuximab and panitumumab are extensively studied and most widely used. Results from multiple Phase III studies showed that the addition of cetuximab or panitumumab to best supportive care or chemotherapy led to improvements in response rates and survival in patients with metastatic colorectal cancer and head and neck cancer [13–18]. However, cetuximab monotherapy is associated with adverse events (AEs) including skin reactions, hypomagnesaemia, mucositis and infusion-related reactions [19]. In particular, skin reactions occurred in the majority of patients (80%) who receive cetuximab [20]. Furthermore, a meta-analysis of the safety of cetuximab in metastatic colorectal cancer found that Grade 3–4 AEs occurred in around 60% of patients, and 90% of patients experienced infusion-related reactions following the first infusion [20].

The development of an anti-EGFR mAb with comparable or higher efficacy to current agents, but with a more favourable safety profile, would be of great clinical utility to patients. In this regard, a novel, recombinant, humanized anti-EGFR antibody HLX07 was designed. As a novel version of anti-EGFR monoclonal antibody, HLX07 was improved in two aspects: first, the Fab portion of HLX07 was re-engineered to modify the glycosylation pattern of this antibody to ensure less immunogenicity and better binding affinity; second, HLX07 was produced in Chinese hamster ovary (CHO) cell system, which led to clearer glycosylation profile and better yield. Since the rare anaphylactic reactions associated with the use of cetuximab is likely related to the specific glycosylation in the molecules and possibly its mouse/human chimeric structure [21], the improvements of HLX07 are hypothesized to produce a safer treatment solution for patients who benefit from anti-EGFR mAb therapy.

In vitro and in vivo data have demonstrated that HLX07 had an anti-cancer effect at least as good as cetuximab at an equivalent dose level. The IC_50_ of HLX07 for the human colorectal carcinoma cell line DiFi and human lung cancer cell line H292 were 54.2 and 23.9 ng/mL (63.7 and 37.2 ng/mL for cetuximab), respectively [22]. In addition, HLX07 had a higher binding affinity to human EGFR versus cetuximab (dissociation constant, 0.143 vs 0.262 nM). Furthermore, HLX07 showed minimal-to-mild toxicity in single-dose and 13-week repeat-dose toxicokinetic studies in *cynomolgus* monkeys at doses up to 60 mg/kg per week. HLX07 is therefore hypothesized to possess improved safety and at least comparable anti-cancer efficacy in patients comparing to current approved anti-EGFR mAbs.

Here, we report the first-in-human, Phase I dose escalation study which aimed to evaluate the safety, tolerability, pharmacokinetics (PK) and preliminary efficacy of HLX07 in patients with advanced solid cancers who had failed standard therapy or for whom no standard therapy was available.

## Methods

### Study design

This was a prospective, open-label, Phase I dose escalation study conducted at three sites in Taiwan. The study followed a traditional “3 + 3” dose escalation design which was described in Supplemental Table 1. HLX07 (Shanghai Henlius Biotech, Inc., China) was administered intravenously (2-h) with an initial dose of 100 mL/h unless a patient developed hypersensitivity reactions. The primary objectives were assessments of the safety and tolerability of HLX07. Secondary objectives included the analysis of PK, evaluation of immunogenicity and investigation of anti-tumor efficacy. The study was conducted following the ethical principles outlined in the Declaration of Helsinki, Council for International Organizations of Medical Sciences (CIOMS) and in line with the International Council on Harmonization Guideline for Good Clinical Practice as well as applicable local regulatory requirements. The study protocol was approved by local ethical review boards before study initiation.

### Patient population

Patients with histologically confirmed metastatic or recurrent epithelial carcinoma who had failed standard therapy or for whom no standard therapy was available were enrolled. Other key inclusion criteria were: white blood cell count ≥3.0 × 10^9^/L, absolute neutrophil count ≥1.5 × 10^9^/L, hemoglobin level > 90.0 g/L platelet count ≥100.0 × 10^9^/L as well as adequate hepatic and renal function. Key exclusion criteria included prior treatment of an anti-EGFR mAb therapy within 3 months before enrollment, any concurrent malignancy other than basal cell carcinoma or carcinoma in situ of the cervix and presence of *K-RAS* or *B-RAF* mutations. A full list of inclusion/exclusion criteria is provided in the Supplemental Table 2. All patients provided written, informed consent before inclusion.

### Endpoints and measurements

The primary study endpoint was a summary listing of patients with treatment-emergent AEs (TEAEs), assessed using the National Cancer Institute Common Terminology Criteria for Adverse Events (NCI CTCAE) version 4.0. Secondary endpoints included PK assessments, overall response rate (ORR) and serum anti-HLX07 antibody assessments.

Safety was evaluated during the whole study period (at screening period, weekly during HLX07 treatment, at the end of treatment visit and during follow-up) by documentation of AEs and serious AEs (SAEs), clinical laboratory investigations, physical examinations, vital sign measurements, 12-lead electrocardiogram (ECG) and World Health Organization performance status (WHO PS). Dose limiting toxicities (DLTs) were assessed for 28 days following the first dose of HLX07.

All patients were included in the PK study. Blood samples were collected prior to and at 1, 2, 5, 24, 72, 96, and 168 h (± 5 min) after the start of the first and forth infusions, before second and third infusions and every eight weeks. The serum concentrations of HLX07 were determined using a validated enzyme-linked immunosorbent assay (ELISA). PK analysis was conducted by WinNonlin (Pharsight Corporation, version 6.3) using the non-compartmental model. The following parameters were calculated: maximum serum concentration (C_max_), time to reach C_max_ (T_max_), serum half-life (T_1/2_), area under the serum concentration-versus-time curve within one dosing interval (AUC_0–168_), area under the serum concentration-versus-time curve until infinity (AUC_0–∞_) and total plasma clearance (CL). C_max_ and T_max_ were derived directly from the serum concentration curve. If timepoints were missing, nominal times could be imputed with sponsor’s approval. Concentrations below the lower limit of quantification, which were before the last quantifiable data point, were taken as zero for calculating the AUC.

Blood samples for immunogenicity assessments were taken before the first and fourth infusions, every eight weeks after the first infusion and at the end of treatment following the same protocol as described for PK blood samples. An electrochemiluminescence immunoassay (ECLIA) was used to detect anti-HLX07 antibodies by Frontage, Inc.

Efficacy endpoints included ORR and two *post-hoc* endpoints: progression-free survival (PFS) and overall survival (OS). Response to treatment was assessed by computed tomography (CT) or magnetic resonance imaging (MRI) per Response Evaluation Criteria in Solid Tumors (RECIST) version 1.1. Imaging assessments were conducted during the screening period (baseline measurement), following the first infusion of HLX07 at 8-week intervals and at the end of treatment visit. Patients who discontinued study treatment before disease progression would be followed until confirmation of disease progression, initiation of subsequent therapy or up to 3 months, whichever occurred first.

### Statistics

The study sample size was based on the expected number of cohorts and patients per cohort. The study planned to enroll a total of 21–36 patients. In this study, the safety population comprised all enrolled patients who received at least one dose of study drug and the PK population included all patients whose plasma concentrations of HLX07 were sufficient to be interpreted. All patients who withdrew prematurely from the study were excluded from the efficacy analysis population and documented along with their primary reasons of withdrawal. All missing data were treated as missing unless otherwise stated.

For the analysis of PFS, patients who were alive and did not have disease progression were censored at the date of the last tumor assessment. For the analysis of OS, patients who had not been reported as a death were censored at the date of the last contact. Kaplan-Meier product limit estimators were used to calculate the 25th, 50th (median) and 75th percentiles of PFS and OS. Results were tabulated with the number of events and censored patients. All analyses were conducted using SAS (Statistical Analysis System, RRID: SCR_008567).

## Results

### Patients

A total of 25 patients were screened and 19 were enrolled between 1 October 2016 and 16 July 2019. Main reasons for screen failure were: meeting exclusion criteria (three), consent withdrawal (two) and presence of *K-RAS* mutations (one). The 19 enrolled patients received weekly administration of HLX07 at doses of 50 mg (n = 3), 100 mg (n = 3), 200 mg (n = 3), 400 mg (n = 3), 600 mg (n = 3) or 800 mg (n = 4, one patient did not complete the study due to sudden death). Patients had a mean age of 58.4 years and a mean body mass index of 23.0 kg/m^2^ (Table 1). The most common cancers included in this study were esophageal carcinoma (26.3%), colorectal cancer (26.3%) and head and neck cancer (21.1%). Most patients (21.1%) had a WHO PS of 0, and 100% had received prior therapy. All patients in the study received concomitant medications, most commonly: aminoalkyl ethers (100%), anilides (100%), glucocorticoids (100%), contact laxatives (63.2%), natural opium alkaloids (57.9%), topical antibiotics (57.9%), benzodiazepine derivatives (52.6%) and propulsives (52.6%).

**Table 1:** Patient baseline characteristics

	HLX07 dose level					
	50 mg (n = 3)	100 mg (n = 3)	200 mg (n = 3)	400 mg (n = 3)	600 mg (n = 3)	800 mg (n = 4)
Mean age, years (SD)	52.3 (14.6)	62.3 (17.1)	59.3 (3.8)	47.3 (9.1)	66.3 (9.6)	61.5 (6.9)
Males, n (%)	3 (100.0)	2 (66.7)	2 (66.7)	3 (100.0)	2 (66.7)	4 (100.0)
Race, Asian, n (%)	3 (100.0)	3 (100.0)	3 (100.0)	3 (100.0)	3 (100.0)	4 (100.0)
Mean weight, kg (SD)	67.4 (8.8)	65.6 (11.0)	61.3 (11.7)	70.8 (12.9)	54.8 (19.8)	64.3 (11.7)
Mean height, cm (SD)	165.3 (3.2)	167.0 (10.8)	163.0 (3.0)	171.7 (7.4)	161.3 (7.1)	168.5 (6.8)
Mean body mass index, kg/m^2^ (SD)	24.8 (4.1)	23.4 (1.0)	23.0 (3.7)	23.9 (3.0)	20.9 (6.8)	22.5 (2.7)
WHO PS, n (%)						
0	0	1 (33.3)	0	0	1 (33.3)	2 (50.0)
1	3 (100.0)	2 (66.7)	3 (100.0)	3 (100.0)	2 (66.7)	1 (25.0)
2	0	0	0	0	0	1 (25.0)
Tumor type, n (%)						
Colorectal cancer	1 (33.3)	2 (66.7)	0	1 (33.3)	1 (33.3)	0
Oesophageal carcinoma	0	0	3 (100.0)	1 (33.3)	1 (33.3)	0
Head and neck	2 (66.7)	0	0	0	1 (33.3)	1 (25.0)
Hepatocellular carcinoma	0	0	0	0	0	3 (75.0)
Pancreatic head carcinoma	0	1 (33.3)	0	0	0	0
Malignant neoplasm of thymus	0	0	0	1 (33.3)	0	0
Tumor stage (TNM), n (%)						
IIIa	0	0	1 (33.3)	0	0	0
IV	1 (33.3)	1 (33.3)	1 (33.3)	3 (100.0)	1 (33.3)	3 (75.0)
IVa	1 (33.3)	1 (33.3)	0	0	0	0
IVb	0	1 (33.3)	1 (33.3)	0	1 (33.3)	0
IVc	1 (33.3)	0	0	0	1 (33.3)	1 (25.0)
Prior lines of therapy, n (%)						
1–2	0	1 (33.3)	1 (33.3)	1 (33.3)	2 (66.7)	1 (25.0)
≥3	3 (100.0)	2 (66.7)	2 (66.7)	2 (66.7)	1 (33.3)	3 (75.0)

*WTO PS* World Health Organization performance status, *SD* standard deviation

### Safety

The majority of patients had good treatment compliance. Seven patients missed ≥1 infusion mostly due to AEs or SAEs. HLX07 was generally well tolerated. 17 patients received >4 doses and tolerated the assigned dose level for 28 days. Two patients did not complete the 4 week treatment: one due to intestinal obstruction and the other due to sudden death. Neither of them were considered related to HLX07. No patient in the study experienced a DLT and the maximum tolerated dose (MTD) was not reached.

All patients included in the study experienced ≥1 TEAE, most commonly fatigue (68.4%), nausea (47.4%), paronychia (31.6%) and vomiting (31.6%) (Table 2). The proportions of patients experienced ≥1 TEAE were similar across all dose cohorts, suggesting there was no association between doses of HLX07 and the toxicities. 11 (57.9%) patients were reported with serious TEAEs (two in 50 mg, three in 100 mg, one in 400 mg, one in 600 mg and four in 800 mg cohort). Among these, only one dyspnea (in 600 mg cohort) was considered possibly related to study treatment.

**Table 2:** Safety data summary

	HLX07 dose level					
n (%)	50 mg (n = 3)	100 mg (n = 3)	200 mg (n = 3)	400 mg (n = 3)	600 mg (n = 3)	800 mg (n = 4)
≥1 TEAE	3 (100.0)	3 (100.0)	3 (100.0)	3 (100.0)	3 (100.0)	4 (100.0)
≥1 serious TEAE	2 (66.7)	3 (100.0)	0 (0)	1 (33.3)	1 (33.3)	4 (100.0)
TEAEs occurring in >10% of total patients						
Fatigue	2 (66.7)	1 (33.3)	2 (66.7)	3 (100.0)	3 (100.0)	2 (50.0)
Nausea	1 (33.3)	0	1 (33.3)	2 (66.7)	3 (100.0)	1 (25.0)
Cough	1 (33.3)	1 (33.3)	1 (33.3)	1 (33.3)	0	2 (50.0)
Paronychia	0	0	2 (66.7)	2 (66.7)	2 (66.7)	0
Vomiting	2 (66.7)	0	0	1 (33.3)	2 (66.7)	1 (25.0)
Constipation	1 (33.3)	0	1 (33.3)	2 (66.7)	1 (33.3)	0
Rash	0	1 (33.3)	2 (66.7)	1 (33.3)	1 (33.3)	0
Acne	0	0	0	2 (66.7)	0	2 (50.0)
Decreased appetite	0	1 (33.3)	0	2 (66.7)	1 (33.3)	0
Headache	1 (33.3)	0	0	2 (66.7)	1 (33.3)	0
Insomnia	1 (33.3)	2 (66.7)	0	0	1 (33.3)	0
Blood lactate dehydrogenase increased	0	0	1 (33.3)	1 (33.3)	1 (33.3)	0
Folliculitis	0	0	1 (33.3)	1 (33.3)	1 (33.3)	0
Hypomagnesaemia	0	0	1 (33.3)	1 (33.3)	1 (33.3)	0
Pyrexia	0	1 (33.3)	0	1 (33.3)	1 (33.3)	0
Asthenia	0	0	0	1 (33.3)	1 (33.3)	0
Blood bilirubin increased	0	1 (33.3)	0	0	0	1 (25.0)
Cellulitis	0	0	0	0	1 (33.3)	1 (25.0)
Dermatitis acneiform	0	0	0	0	1 (33.3)	1 (25.0)
Dehydration	0	1 (33.3)	0	1 (33.3)	0	0
Diarrhea	0	0	1 (33.3)	1 (33.3)	0	0
Dizziness	0	0	0	1 (33.3)	1 (33.3)	0
Dyspnea	1 (33.3)	0	0	0	1 (33.3)	0
Hematuria	0	0	1 (33.3)	1 (33.3)	0	0
Hypertension	1 (33.3)	0	0	1 (33.3)	0	0
Hypoesthesia	0	0	0	1 (33.3)	1 (33.3)	0
Hypoglycemia	0	1 (33.3)	0	0	1 (33.3)	0
Mucosal inflammation	1 (33.3)	0	0	0	1 (33.3)	0
Myalgia	0	0	0	2 (66.7)	0	0
Neutrophil count decreased	0	0	0	1 (33.3)	0	1 (25.0)
Pleural effusion	1 (33.3)	0	0	1 (33.3)	0	0
Productive cough	0	0	1 (33.3)	1 (33.3)	0	0
Pruritus	1 (33.3)	0	1 (33.3)	0	0	0
Sepsis	1 (33.3)	1 (33.3)	0	0	0	0
Sinus tachycarida	1 (33.3)	0	0	1 (33.3)	0	0
Skin fissures	0	0	0	0	1 (33.3)	1 (25.0)
Thrombocytopenia	1 (33.3)	0	0	0	0	1 (25.0)
Weight decreased	0	1 (33.3)	0	0	1 (33.3)	0

*TEAE* treatment-emergent adverse events

At the time of final analysis, all patients had discontinued study treatment due to tumor progression (57.9%), AEs (26.3%), investigator’s discretion (10.5%) and withdrawal of consent (5.3%). The five patients who discontinued treatment due to AEs were from 50 mg (one with leukopenia and sepsis), 100 mg (one with intestinal obstruction) and 800 mg cohort (three due to sudden death, metastasis to the central nervous system and ruptured tumor, respectively). None of these AEs were considered related to HLX07. A total of eight patients died during the study (one in 50 mg, two in 100 mg, one in 400 mg, one in 600 mg and three in 800 mg cohort). Five deaths were due to TEAEs, but none were related to HLX07.

### Pharmacokinetic properties

**Fig. 1:**
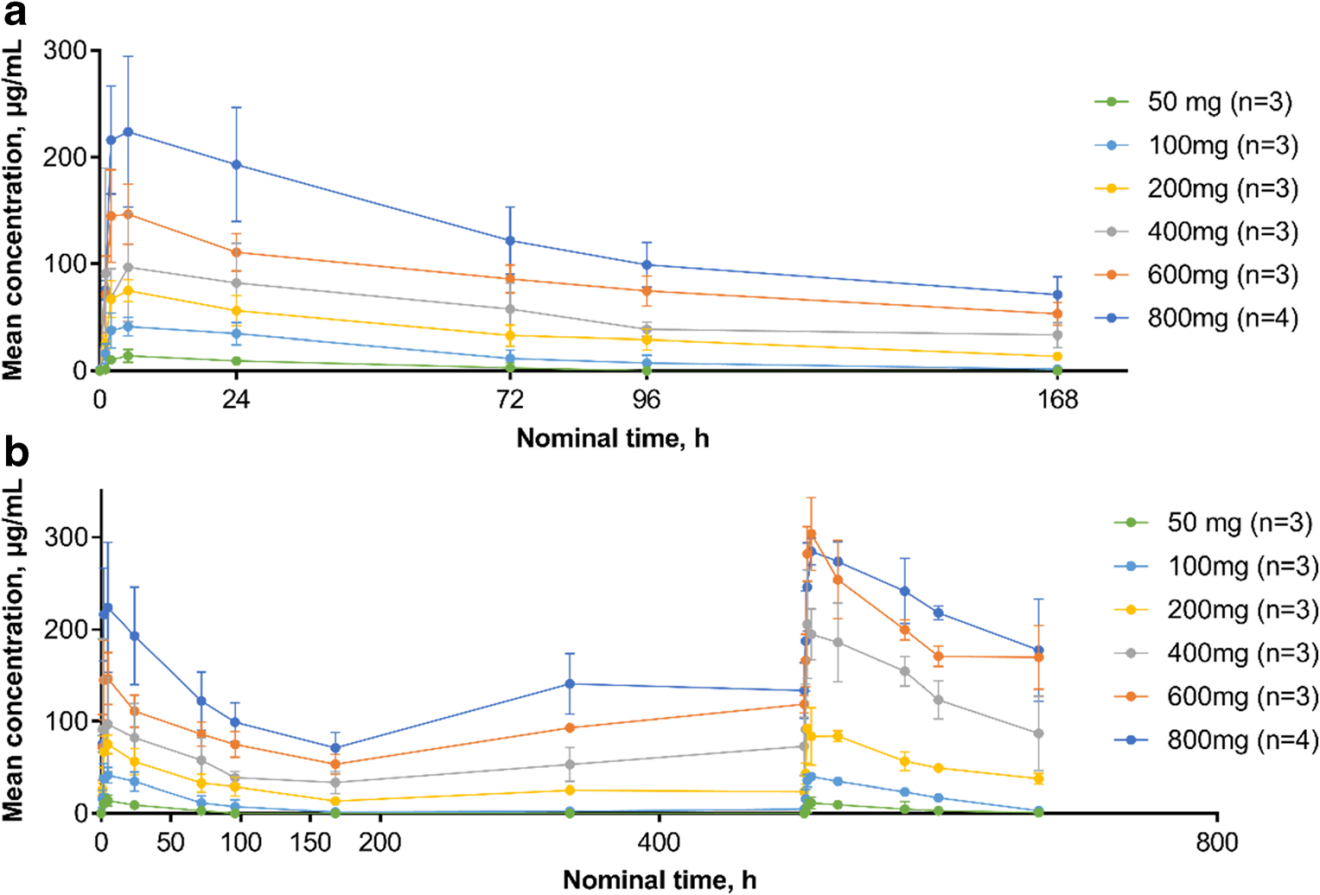
Mean serum concentration-time profiles following a single **a**) and multiple **b**) infusions of HLX07. Linear scale, bars represent standard deviation

**Fig. 2:**
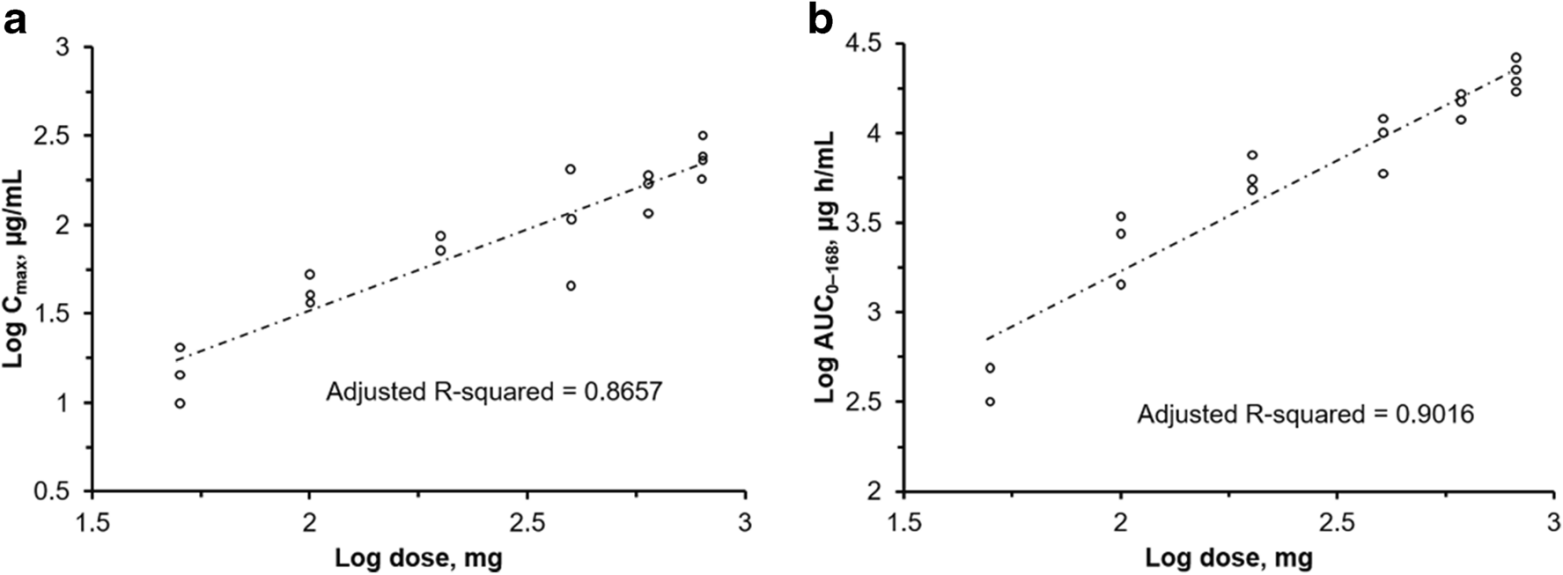
Relationship between systemic exposures to HLX07 indicated by **a**) C_max_ and **b**) AUC_0–168_ and dose (50 to 800 mg) following a single dose infusion (log-log plots). AUC_0–168_, area under the serum concentration-time curve from time zero to 168 h post start of infusion; C_max_, maximum serum concentration

The PK analysis included all 19 patients. Mean serum concentration time curves following single and multiple doses of HLX07 revealed dose dependent increases in serum concentrations (Fig. 1a and b). A summary of the HLX07 PK properties is provided in Table 3. Median T_max_ ranged between 1.9 and 5.0 h across the dose cohorts. Mean values of C_max_ and AUC_0–∞_ in the different dose cohorts indicated that systemic exposure to HLX07 increased proportionally with dose (Fig. 2a and b). CL appeared to be constant across all dose levels.

**Table 3:** HLX07 pharmacokinetic parameters following a single dose infusion

Dose, mg	AUC_0–168_ (μg h/mL) [CV%]	AUC_0–∞_ (μg h/mL) [CV%]	C_max_ (μg/mL) [CV%]	Median T_max_ (h) (range)	CL (mL/h) [CV%]	T_1/2_ (h) [CV%]
50 (n = 3)	401.7 [31.4]	–	15.0 [36.8]	2.1 (2.0–5.1)	130.9 [31.4]	–
100 (n = 3)	2542.0 [41.8]	3316.4 [21.1]	43.2 [20.4]	5.0 (2.0–24.0)	45.7 [50.9]	38.4 [31.1]
200 (n = 3)	5914.3 [25.3]	7350.1 [22.0]	76.1 [11.8]	5.0 (2.1–5.1)	35.2 [23.1]	75.1 [8.3]
400 (n = 3)	9174.3 [34.3]	34,057.3 [110.6]	118.7 [68.2]	1.9 (1.1–5.0)	47.9 [40.0]	411.2 [133.9]
600 (n = 3)	14,279.7 [17.3]	25,036.3 [22.3]	157.7 [24.4]	5.0 (2.0–5.0)	42.9 [18.6]	138.7 [10.5]
800 (n = 4)	21,059.2 [19.8]	36,513.7 [23.8]	234.0 [24.2]	5.0 (5.0–24.0)	39.1 [19.4]	148.0 [37.7]

All values are mean unless otherwise stated; − indicates HLX07 dose below the level of detectability

AUC_0–168_, area under the serum concentration-time curve from time zero to 168 h post start of infusion; AUC_0–∞_, area under the serum concentration-time curve from time zero to the time of the last measurable concentration; CL, clearance; C_max_, maximum serum concentration; CV%, coefficient of variation; T_1/2_, serum half-life; T_max_, time of the maximum serum concentration

A comparison of systemic exposure to HLX07 following the first and fourth infusions revealed that accumulation of HLX07 was negligible at lower doses (50–200 mg). However, at higher doses (400–800 mg), systemic exposure to HLX07 was higher following the fourth infusion versus the first infusion, suggesting accumulation of HLX07 in this dose range (Supplemental Table 3).

### Immunogenicity

Only one patient exhibited a low level of anti-HLX07 antibodies at the first week pre-dose. All other patients were seronegative for anti-HLX07 antibodies at each of the assessed timepoints.

### Treatment efficacy

Of the 19 enrolled patients, 16 were evaluable for treatment response. No patients achieved a complete response (CR). One (5.3%) patient in 600 mg dose cohort with advanced colon cancer, who had received rituximab previously, achieved a partial response (PR). Five patients (26.3%) had stable disease (SD) and ten (52.6%) experienced progressive disease (PD) (Supplemental Fig. 1). All patients who achieved SD or PR received HLX07 for >75 days. Among all patients, the median PFS was 1.9 months (95% CI, 1.7–3.6). For OS analysis, 8 patients had events and 11 were censored under 12-week post-treatment follow-up period. The median overall survival time was not estimable by current data, while the 25th percentile of OS was 2.17 months.

## Discussion

Anti-EGFR mAbs are established treatments for patients with metastatic colorectal and head and neck cancer [9–12]. However, the currently available anti-EGFR mAbs are associated with a relatively high incidence of AEs including skin reactions and electrolyte disorders [20]. HLX07 is a novel anti-EGFR antibody developed with a re-engineered antigen-binding fragment, aiming to enhance its binding affinity and safety.

The results of this first-in-human Phase I study showed that weekly administration of HLX07 up to 800 mg was well tolerated, with no unexpected safety signals. Furthermore, the safety profile of HLX07 was comparable across all dose cohorts which indicated no relationship between weekly administration of HLX07 and incidence of AEs. The most frequently observed TEAEs in this study were consistent with the expected safety profile of anti-EGFR mAbs [20]. Fatigue (68.4%), nausea (47.4%), paronychia (31.6%) and vomiting (31.6%) reported in this study were mainly Grade 1 or 2. Acne and hypomagnesemia are frequently experienced by patients receiving anti-EGFR mAbs, and incidence rates of these AEs for patients receiving HLX07 were 21.1% and 15.8%, respectively. Of the 11 patients who experienced a serious TEAE, only one (dyspnea) was considered possibly related to HLX07 treatment.

This study provided the first-in-human description of the PK properties of HLX07. The PK data showed that maximum HLX07 serum concentration was generally reached around three hours after the end of infusion. The mean C_max_ and AUC_0–∞_ across the dose cohorts indicated that systemic exposure to HLX07 increased with dose, which was consistent with PK profiles reported for cetuximab and panitumumab [23, 24]. In addition, accumulation of HLX07 between the first and fourth infusions was observed among patients receiving 400–800 mg/week doses. Valuable initial insights were provided by this study, but the relatively small patient number in each dose cohort suggested that the mean values of PK parameters were sensitive to inter-patient variability and outliers. Thus, further investigation of HLX07 in larger studies is necessary.

This study was not designed to assess treatment efficacy. Indeed, as a dose escalation study, it would be expected that optimal HLX07 dosing would not be received by all patients. Additionally, anti-EGFR mAbs are usually administered in combination with chemotherapy for maximum efficacy. Despite these, weekly HLX07 monotherapy led to SD in five patients and PR in one patient with advanced colon cancer (600 mg). In addition, all patients who achieved SD or PR received HLX07 for >75 days, which may further suggest the efficacy of HLX07 monotherapy.

This study had several potential limitations. Firstly, no patient in the study experienced a DLT, and the MTD was not identified. Secondly, this study investigated HLX07 monotherapy, but in real clinical practice anti-EGFR mAbs are often combined with chemotherapy. Finally, patients included in this study had very advanced and heavily pre-treated disease which may have influenced the incidence of AEs and not be representative for the safety of HLX07 in patients with less advanced disease.

In conclusion, this first-in-human trial showed that HLX07 monotherapy was well tolerated (at doses up to 800 mg) and promising in patients with advanced solid tumors. The findings of this study support the initiation of a Phase Ib/II clinical study of HLX07 combined with chemotherapy in patients with advanced solid tumors (NCT03577704).

## Supplementary Information

ESM 1 (PDF 375 kb)

## Data Availability

The data that support the findings of this study are available from the corresponding author upon reasonable request.
