# Supplementary material for: Phase I first-in-human study of HLX07, a novel and improved recombinant anti-EGFR humanized monoclonal antibody, in patients with advanced solid cancers
Source: Invest New Drugs. 2021 Mar 13;39(5):1315–23. doi: 10.1007/s10637-021-01099-1 (PMC8426222; doi:10.1007/s10637-021-01099-1)
Supplement: Supplementary file 1 — (PDF 375 kb) [file 10637_2021_1099_MOESM1_ESM.pdf]

Supplemental Table 1. Dose escalation scheme

Number of patients with dose-limiting toxicity at a given dose level	Escalation decision
0/3	● Escalate to next higher dose level for a new cohort of three patients
1/3	● Enter ≥3 additional patients at the same dose level<br>● If 0/3 of the additional patients experience a DLT (1/6), proceed to the next dose level<br>● If ≥1/3 of the additional patients experience a DLT (2/6), cease escalation and consider this the maximal administered dose<br>○ Expand the previous dose cohort to six patients and if < 1/6 experience a DLT, this dose is the MTD
≥2/3	● Cease dose escalation and this dose will be considered the maximal administered dose<br>○ Expand the previous dose cohort to six patients and if < 1/6 experience a DLT, this dose is the MTD
≤1/6 at the highestdose level below themaximal administered dose	● This will be defined as the recommended phase 2 dose<br>○ ≥6 patients should have been enrolled at this dose level to determine the recommended phase 2 dose

Supplemental Table 2. Inclusion and exclusion criteria

**Inclusion criteria**	
**1**	Patients aged 18 years or more with histologically-confirmed, unidimensionally-measurable and/or evaluable metastatic or recurrent epithelial carcinoma which had failed standard therapy or for whom no standard therapy was available. (for patients in Taiwan, age ≥20).
**2**	World Health Organization (WHO) performance status score of ≤2 at study entry.
**3**	Able to provide written informed consent.
**4**	White blood cell (WBC) count ≥3 × 10^9/L, absolute neutrophil count ≥1.5 × 10^9/L, haemoglobin level >90 g/L and platelet count ≥100 × 10^9/L.
**5**	Adequate hepatic function (alkaline phosphatase ≤ 5.0 × Upper Limit Of Normal (ULN), bilirubin ≤1.5 × ULN, aspartate transaminase (AST) and alanine transaminase (ALT) ≤2.5 × ULN or ≤5 × ULN for patients with liver metastases.
**6**	Adequate renal function as defined by a serum creatinine level within normal limits.
**7**	Use of effective contraception if procreative potential existed.
**8**	Life expectancy ≥3 months as assessed by the investigators.
**Exclusion criteria**	
**1**	Chemotherapy, radiation, monoclonal antibody therapy (except for anti-EGFR monoclonal antibody) and/or hormonal therapy (except palliative radiation therapy for disease-related pain and chronic hormonal therapy for prostate carcinoma) less than 4 weeks of the first infusion of HLX07 (for prior anti-EGFR antibody therapy, refer to Exclusion Criteria 8).
**2**	Concurrent unstable or uncontrolled medical disease (e.g., active uncontrolled systemic infection, poorly controlled hypertension or history of poor compliance with an anti-hypertensive regimen, unstable angina, congestive heart failure, uncontrolled diatetes) or other chronic disease, which, in the opinion of the investigator, could compromise the patient or the study.
**3**	Newly-diagnosed or symptomatic brain metastases (patients with a history of brain metastases must have received definitive surgery or radiotherapy, be clinically stable, and not taking steroids; anticonvulsants were allowed).
**4**	Any concurrent malignancy other than basal cell carcinoma or carcinoma in situ of the cervix. Patients with a previous malignancy but without evidence of disease for more than 3 years would be allowed to enter the trial.
**5**	Any condition that prevented the patient from providing informed consent.
**6**	Pregnancy (confirmed by urine beta human chorionic gonadotropin [beta-HCG]) or breastfeeding.
**7**	Any investigational device(s) within 4 weeks of the first infusion of HLX07.
**8**	Prior treatment with cetuximab, or any other anti-EGFR monoclonal antibody therapy for less than 3 months. Prior treatment with other monoclonal antibodies targeting receptors other than the EGFR was permitted if the drug had been discontinued ≥4 weeks prior to the first infusion of HLX07.
**9**	Tumor cells with either *K-RAS* or *B-RAF* mutations.
**10**	Known history of human immunodeficiency virus infection.
**11**	Employees of the investigator or study center with direct involvement in this study or other studies under the direction of the investigator or study center, as well as family members of the employees.

Supplemental Table 3. Systemic exposure to HLX07 following the first and fourth doses

	C_{max}, µg/mL[a] (CV%)			$AUC_{0–168}$, µg h/mL[a] (CV%)		
**Dose, mg**	**1st infusion**	**4th infusion**	**Ratio[b]**	**1st infusion**	**4th infusion**	**Ratio[b]**
**50 (n = 3)**	15.0 (36.8)	14.8 (19.2)	0.98	401.7 (31.4)	497.8 (31.6)	1.24
**100 (n = 3)**	43.2 (20.4)	40.6 (1.7)	0.94	2542.0 (41.8)	3245.0 (14.0)	1.28
**200 (n = 3)**	76.1 (11.8)	98.5 (5.7)	1.29	5914.3 (25.3)	9617.8 (10.0)	1.67
**400 (n = 3)**	118.7 (68.2)	216.2 (19.6)	1.82	9174.3 (34.3)	23516.2 (9.7)	2.56
**600 (n = 3)**	157.7 (24.4)	311.1 (11.2)	1.97	14279.7 (17.3)	34133.3 (3.8)	2.39
**800 (n = 4)**	240.0 (24.2)	285.3 (5.1)	1.19	21059.2 (19.8)	37665.9 (8.2)	1.79

[a]All values are mean (SD); [b]Ratio = 4th value/1st value.

$AUC_{0–168}$, area under the serum concentration-time curve from time zero to 168 hours post start of infusion; C_{max}, maximum serum concentration; CV%, coefficient of variation.

Supplemental Fig. 1. Summary of tumor responses to HLX07

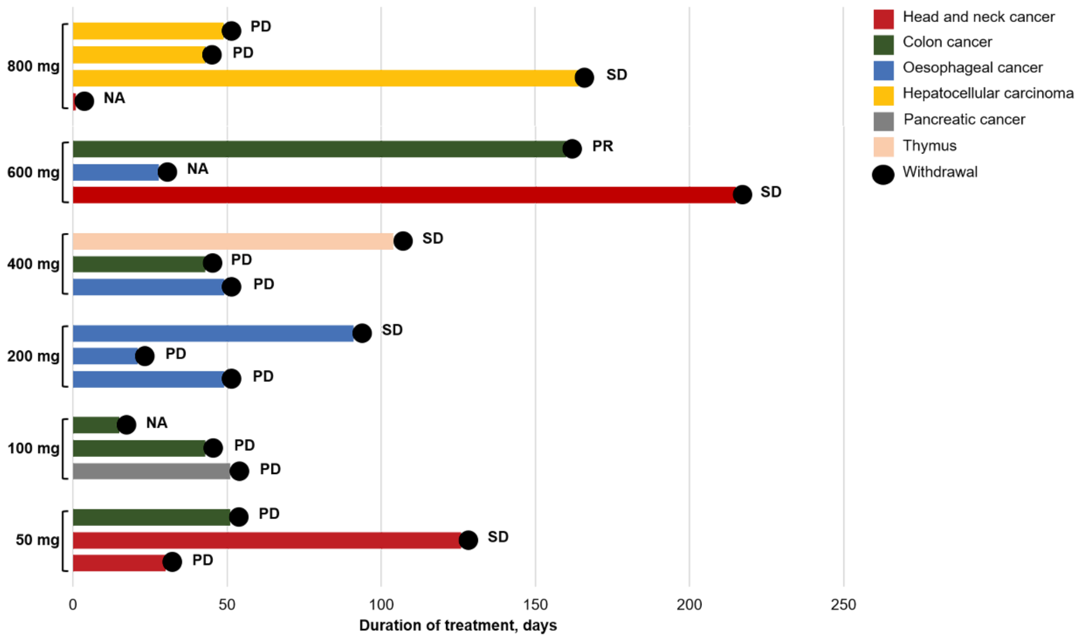

NA, not available; PD, progressive disease; PR, partial response; SD, stable disease.
